# The oncogenic roles and clinical implications of YAP/TAZ in breast cancer

**DOI:** 10.1038/s41416-023-02182-5

**Published:** 2023-02-09

**Authors:** Juan Luo, Hailin Zou, Yibo Guo, Tongyu Tong, Yun Chen, Yunjun Xiao, Yihang Pan, Peng Li

**Affiliations:** 1 Scientific Research Center, The Seventh Affiliated Hospital of Sun Yat-sen University, No. 628 Zhenyuan Road, 518107 Shenzhen, Guangdong People’s Republic of China; 2 Department of Urology, Pelvic Floor Disorders Center, The Seventh Affiliated Hospital of Sun Yat-sen University, No. 628 Zhenyuan Road, 518107 Shenzhen, Guangdong People’s Republic of China; 3 Guangdong Provincial Key Laboratory of Digestive Cancer Research, The Seventh Affiliated Hospital of Sun Yat-sen University, No. 628 Zhenyuan Road, 518107 Shenzhen, Guangdong People’s Republic of China

**Keywords:** Breast cancer, Breast cancer

## Abstract

Breast cancer (BC) is the most commonly diagnosed form of cancer and a leading cause of cancer-related deaths among women worldwide. Yes-associated protein (YAP) and transcriptional coactivator with PDZ-binding motif (TAZ) are homologous transcriptional coactivators and downstream effectors of Hippo signalling. YAP/TAZ activation has been revealed to play essential roles in multiple events of BC development, including tumour initiation, progression, metastasis, drug resistance and stemness regulations. In this review, we will first give an overview of YAP/TAZ-mediated oncogenesis in BC, and then systematically summarise the oncogenic roles of YAP/TAZ in various BC subtypes, BC stem cells (BCSCs) and tumour microenvironments (TMEs). Based on these findings, we will further discuss the clinical implications of YAP/TAZ-based targeted therapies in BC and the potential future direction.

## Introduction

**Fig. 1:**
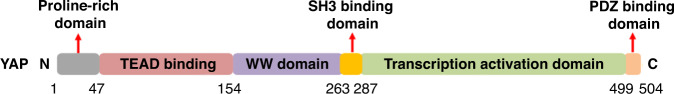
Schematic overview of the basic structure of YAP protein. YAP protein is mainly composed of six domains (from N- to C-terminus): proline-rich region, TEAD-binding domain, WW domain, SH3-binding domain, transcription activation domain, and the PDZ-binding domain.

Yes-associated protein (YAP) was initially discovered by Sudol in chicken as a novel protein binding to the Src homology 3 (SH3) domain of tyrosine kinases, including YES, SRC and ABL [1]. Subsequently, mouse and human homologues were identified and named YAP 65 for Yes-associated protein of 65 kDa [2]. This protein is encoded at the 11q22 locus and consists of six domains from N- to C-terminus: proline-rich region, TEAD-binding domain, WW domain, SH3 binding domain, transcription activation domain and the PDZ-binding domain (Fig. 1) [3]. Shortly thereafter, YAP was shown to function as a transcriptional co-activator that was usually recruited by other transcription factors to coordinate the transcriptional regulation [4]. So far, extensive studies have confirmed that YAP predominantly uses the TEAD family of transcription factors to execute its functionally relevant transcription programs [5]. Transcriptional coactivator with PDZ-binding motif (TAZ) was first identified as a novel 14-3-3-binding protein, and then proved to be an evolutionarily conserved paralog of YAP in mammalian cells [6]. TAZ shares nearly 50% sequence similarity and the same overall structural organisation with YAP [3]. In addition, it also functions as a transcriptional co-activator to participate in the transcriptional gene regulation.

**Fig. 2:**
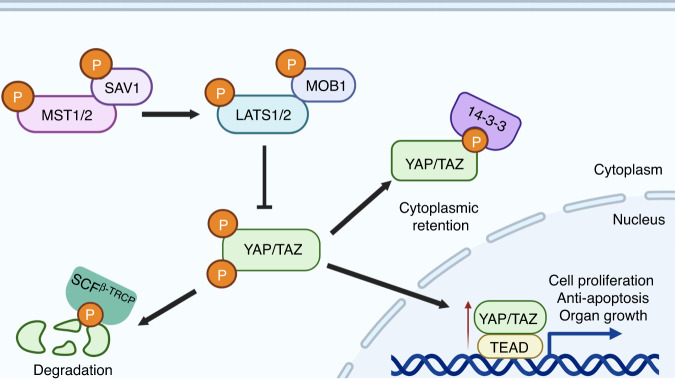
The core components of Hippo-YAP/TAZ signalling and Hippo kinase-mediated YAP/TAZ regulations. The core components of this signalling include the core kinase cascade (MST1/2 and LATS1/2), adaptors (SAV, MOB1A/B) and the downstream effectors YAP/TAZ. When the kinase cascade is activated, LATS1/2 operate an inhibitory phosphorylation of YAP/TAZ, leading to their cytoplasmic retention and proteasomal degradation. When the Hippo kinase cascade is inactivated, YAP/TAZ can translocate to the nucleus where they interact with TEADs (the main transcriptional partners) to induce the transcription of downstream target genes, thereby promoting cell proliferation, anti-apoptosis and organ growth.

With the discovery and functional characterisation of the Hippo pathway kinase cassette in mammals, including STE20-like kinases 1/2 (MST1/2), adaptor protein Salvador 1 (SAV1), large tumour suppressor 1/2 (LATS1/2) and MOB kinase activator 1A/B (MOB1A/B) [7, 8], YAP/TAZ are further identified to be the major downstream effectors of this pathway [9]. Functionally, Hippo pathway converges on YAP/TAZ to regulate cell proliferation, apoptosis and organ growth (Fig. 2) [10–13]. Mechanistically, MST1/2 kinase can complex with SAV1 to directly phosphorylate MOB1 and LATS1/2, which is required for LATS activation and an increased LATS kinase activity [14]. LATS activation in turn phosphorylates YAP at multiple serine residues, including Ser 61, 109, 127, 164 and 381 [15]. Among these residues, Ser 127 and Ser 381 are the key phosphorylation sites in suppressing YAP activity. Specifically, YAP phosphorylation at Ser 127 results in its binding by 14-3-3 protein and cytoplasmic retention, thereby spatially isolating YAP from its nuclear target transcription factors, like TEADs [15]. Phosphorylation of YAP at Ser 381 will facilitate its subsequent phosphorylation by CK1 kinase and recognition by SCF^β-TRCP^ E3 ubiquitin ligase, which eventually induce YAP degradation (Fig. 2) [15]. Therefore, either inactivation of the Hippo kinases or YAP/TAZ constitutive activation can induce the cell hyperproliferation and organ overgrowth through promoting YAP/TAZ nucleus accumulation and downstream gene transcription [10, 13, 16–18].

In the past two decades, accumulating evidence has revealed that YAP/TAZ are central hubs of complex cell and organ regulations. They coordinate the mechanical, metabolic, extracellular and intracellular signals to regulate many physiological and pathological processes, including development, tissue homoeostasis and regeneration, fibrosis, inflammation, and tumourigenesis. Especially in human cancers, loss-of-function (LOF) mutations in negative regulators of YAP/TAZ or activating mutations that have the potential to promote nuclear YAP/TAZ activity have been observed. These cancer-associated upstream regulators include MST1/2, LATS1/2, SAV1, NF2, PTPN14, FAT1, GNAQ/GNA11, SRC, etc. [19, 20]. Therefore, targeting these upstream regulators to block the activity of YAP/TAZ offers a potential opportunity for tumour therapy.

## Overview of YAP/TAZ-mediated oncogenesis in BC

BC is the most common type of malignant cancer among women around the world, and also ranks as first in terms of cancer-related deaths [21]. BC usually originates from the epithelial cells lining the ducts of the breast. On the basis of origin sites, BC is divided into ductal carcinoma, lobular carcinoma, and other types of carcinomas [22]. In addition, according to its growth ability, BC includes carcinoma in situ and invasive carcinoma [22]. As a key regulator of Hippo-YAP/TAZ signalling for cell proliferation and organ growth, current research has revealed that YAP/TAZ activation is involved in multiple events through tumourigenesis and development in BC. These include tumour growth, metastasis, drug resistance, TME regulation, angiogenesis and cancer stemness regulation [22]. In 2006, Overholtzer et al. initially reported that overexpression of human YAP in nontransformed mammary epithelial cells (MCF10A) induced epithelial-to-mesenchymal transition (EMT), growth factor-independent proliferation, and anchorage-independent growth in soft agar [23]. In 2011, Cordenonsi et al. first showed that TAZ was required to sustain the self-renewal and tumour-initiation capacities of BCSCs [24]. Subsequent studies confirmed that YAP/TAZ-induced phenotypes were negatively regulated by Hippo kinase cassette and dependent on their interactions with TEAD family transcription factors [10, 13, 15, 25–32]. Meanwhile, multiple YAP/TEAD downstream target genes, including *CTGF*, *CYR61*, *AREG* and the mitotic genes, were identified to be required for YAP/TAZ-dependent epithelial cell proliferation and transformation [33, 34]. Later, Li et al. found that constitutive activation of YAP (S127A) in mouse mammary stem cells (MaSCs) caused rapid formation of large tumours in an orthotopic allograft mouse model [35]. Similarly, Panciera et al. also showed that transient upregulation of exogenous YAP/TAZ in primary mammary epithelial cells could efficiently reprogramme them into MaSC-like cells [36]. These cells exhibited molecular and functional properties similar to endogenous MaSCs, including organoid formation and mammary gland reconstitution after transplantation [36]. Taken together, all these studies in cell model indicate a potential oncogenic role for YAP/TAZ in mammary cell transformation and tumour formation.

Despite these tumour-promoting phenotypes of YAP/TAZ in vitro, their in vivo functions seem to be more moderate. Hyperactivation of YAP in mouse mammary epithelia did not induce hyperplasia, but caused defects in terminal differentiation [37]. Meanwhile, forced expression of TAZ in luminal cells induced them to adopt basal characteristics, as well as an increase in mammary glandular size and weight; however, it was not able to induce the mammary tumour initiation [38, 39]. These studies thus demonstrate that YAP or TAZ activation alone is not sufficient to induce the mammary tumour initiation. Nonetheless, emerging evidence has revealed that YAP/TAZ activity is required for the mammary tumour progression and metastasis. For example, inactivation of YAP suppressed the mammary tumour growth and metastasis caused by either *PyMT* activation or *Apc* loss [37, 40], while TAZ activation dramatically accelerated the DMAT-induced mammary tumour formation [39]. In addition, a recent study showed that deletion of *Mob1a/b* in mouse mammary luminal epithelium induced rapid and highly reproducible mammary tumourigenesis, and which was dependent on TAZ activity [41]. *TP53* is one of the most frequently mutated genes in BCs, and *p53* loss can induce the mammary tumourigenesis from luminal cells [42, 43]. Tao et al. found that YAP expression was dramatically elevated in *p53* loss-driven mammary tumours from luminal cells, indicating a potential cooperation between *Yap* overexpression and *p53*-loss in BC development [44]. Besides, YAP activation was also found to be required for t-ASPP2-induced invasive lobular carcinoma (ILC) growth and progression [45]. Taken together, all these findings highlight the key roles of YAP/TAZ in both breast epithelial lineage commitment, and BC progression and metastasis.

## YAP/TAZ-mediated oncogenesis in BC subtypes

Based on the expression levels of estrogen receptor (ER), progesterone receptor (PR) and human epidermal growth factor 2 (HER2) protein, BC is divided into three categories: luminal, HER2-positive (HER2+), and triple-negative BC (TNBC: ER−/PR−HER2−) subtypes. Among them, luminal and HER2+ subtypes respond sensitively to hormone receptor (HR)- and HER2-targeted therapy respectively, while the TNBC subtype usually lacks effective targeted therapy and thus shows a relatively poor prognosis [46]. In recent years, multiple targeted drugs have also been approved by FDA for the treatment of BC, including inhibitors of cyclin-dependent kinase 4/6 (CDK4/6), mTOR, poly (ADP-ribose) polymerase (PARP), and epithelial growth factor receptor (EGFR) [46]. However, BC subtypes still show different responses to systemic therapy. The main underlying reasons include natural or acquired drug-resistance, and the lack of reliable indicators for most of the targeted agents [46]. Therefore, identifying novel BC therapeutic targets is still essential for developing patient-specific treatment strategies. Considering the increasingly important roles of YAP/TAZ in BC, in this part, we attempt to systematically summarise and discuss the YAP/TAZ-mediated oncogenesis in various BC subtypes.

### Luminal subtype

Luminal subtype of BC is typically ER positive (ER+) and represents around 70% of total BC [47]. ER has two different subtypes, ERα and ERβ, which belong to the superfamily of nuclear HR and act as a transcription factor regulated mainly by oestrogen [47]. Between these two receptors, ERα is encoded by *ESR1* and essential for the tumourigenesis and malignancy of luminal subtype of BC. Estrogen derivation and ERα antagonists have been widely used for the BC patient treatment with ER expression, which have also greatly improved patient outcomes and survival [47]. However, drug resistance still persists in most of the patients after prolonged treatment.

**Fig. 3:**
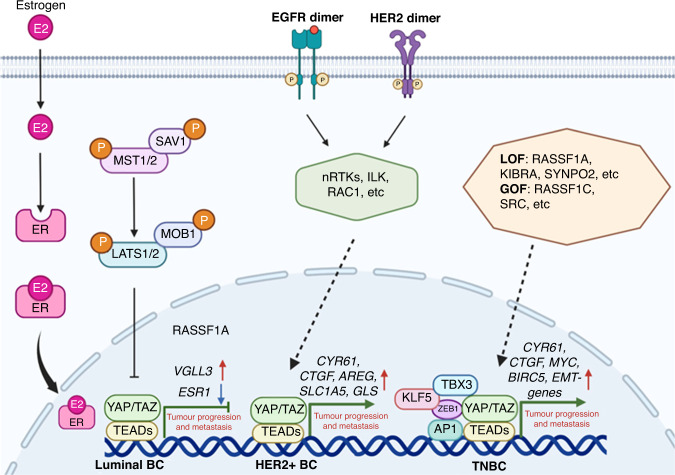
Schematic overview of the molecular mechanisms of YAP/TAZ in BC progression and metastasis. In luminal BC, YAP/TAZ function as a tumour suppressor by disrupting a TEAD-ERα signalling axis; in HER2+ BC, YAP/TAZ activation is responsible for the transcriptional responses downstream of oncogenic signalling, including HER2, EGFR, etc; In TNBC, YAP/TAZ-TEAD complex cooperates with other transcription factors to synergically promote the oncogenic growth and metastasis, including AP1, ZEB1, KLF5, TBX3, etc. E2: 17β-estradiol, nRTK: non-receptor tyrosine kinase.

YAP/TAZ have been reported to be involved in the transcriptional regulation of *ESR1* gene, as well as ERα-associated tumourigenesis (Table 1 and Fig. 3). The early study by Zhou et al. showed that estrogen could activate YAP/TAZ activity through G protein-coupled oestrogen receptor (GPER) independently of ERα, thereby modulating breast epithelial cell proliferation [48]. This study firstly linked the YAP/TAZ activity with estrogen/GPER-mediated breast tumourigenesis. Subsequently, Britschgi et al. reported that YAP/TAZ could cooperate with ERα and thereby promote the luminal phenotype and increase the number of bipotent and luminal progenitors [49], indicating a cooperative role between YAP/TAZ and ERα in the regulation of breast cell fate. Further study by Zhu et al. revealed that YAP/TEAD non-canonically bound to a group of ERα-bound enhancers, and which was required for estrogen-induced transcription and BC growth [50]. Based on the above evidence, YAP/TAZ definitely exert a tumour-promoting function in ER+ BC through enhancing both estrogen-mediated signalling transduction and ERα-associated transcriptions. However, several recent studies have provided conflicting results regarding the function and regulatory mechanism of YAP/TAZ in ER+ BC. For example, Ma et al. have shown that YAP/TAZ directly repress *ESR1* gene transcription, thereby inhibiting the growth of ER+ BC cells [51]. Further investigations revealed that YAP-TEAD module stimulated the transcription of vestigial-like protein 3 (*VGLL3*), which in turn competed with YAP/TAZ for binding to TEAD transcription factor and then recruited the NCOR2/SMRT repressor to the super-enhancer of *ESR1* gene, leading to epigenetic alteration and its transcriptional silencing [52]. Alternatively, Li et al. showed that TEAD also physically interacted with ERα to promote ERα binding to its target promoters/enhancers, thereby inducing the transcription of *ESR1* [53]. On the contrary, YAP competed with ERα for binding to TEAD, leading to decreasing the DNA-binding of ERα and blocking its transcription [53]. These studies thus highlight that YAP/TAZ function as tumour suppressors in ER+ BC. Considering the multifarious and paradoxical roles of YAP/TAZ, cautions should be taken in clinical trials by targeting the Hippo-YAP/TAZ signalling in luminal subtype of BC.

**Table 1:** YAZ/TAZ-mediated oncogenesis in BC.

Cell types	Context	Partners	Transcriptional outputs	Functions	Main reference
Mammary epithelium	MCF10A	TEADs	YAP/TEAD target genes	YAP is required for mammary epithelial cell transformation, oncogenic growth and metastasis.	[10, 13, 15, 23, 25–27, 32]
	MCF10A	TEADs	TAZ/TEAD target genes	TAZ is required for mammary epithelial cell transformation, oncogenic growth and metastasis, as well as BC stemness and chemoresistance.	[24, 28–32]
	MCF10A	TEADs	*AREG*	YAP-induced *AREG* expression is required for YAP-mediated cell proliferation and migration, but not EMT.	[33]
	MCF10A	TEADs and B-MYB	B-MYB and mitotic genes	YAP and B-MYB are critical for YAP-mediated entry into mitosis.	[34]
	Mouse mammary stem cells	/	/	YAP activation in mouse MaSCs causes rapid formation of large tumours in an orthotopic allograft mouse model.	[35]
	Mammary epithelial cell reprogramming	/	/	Transient upregulation of exogenous YAP/TAZ in primary mammary epithelial cells can efficiently reprogramme them into MaSC-like cells.	[36]
	*PyMT*-induced mammary tumourigenesis	/	/	YAP is required for the terminal differentiation of mammary epithelia, and *PyMT*-induced mammary tumour growth.	[37]
	Breast epithelial lineage commitment	SWI/SNF	Luminal and basal cell-specific genes	TAZ/SWI/SNF complex can repress the expression of luminal cell-specific genes and activate basal cell-specific genes.	[38]
	DMBA-induced mammary tumourigenesis	/	/	TAZ regulates mammary gland morphogenesis and carcinogen-induced mammary tumourigenesis.	[39]
	*Apc* loss-induced mammary tumourigenesis	TEADs and BRD4	Growth-regulating genes	YAP/TAZ and BRD4-mediated transcription is responsible for the transcriptional addiction in BC.	[40]
	*Mob1a/b* knockout-induced mammary tumourigenesis	TEADs	*KRT14*, *KRT5*, *EGFR*, and *KRT17*	TAZ is activated in human premalignant BLBC and a key driver of human BLBC.	[41]
	*p53* loss-induced mammary tumourigenesis	/	/	YAP overexpression is required for *p53* loss-induced tumourigenesis.	[42–44]
	t-ASPP2-associated ILC	/	/	YAP activation induced by truncation variant of ASPP2 contributes to tumour growth and progression.	[45]
Luminal BC	GPER-mediated BC	TEADs	*CTGF*, *CYR61*, *EDN1*, and *EGR1*	YAP/TAZ is required for GPER-induced gene transcription, BC cell proliferation and migration, and tumour growth.	[48]
	*Lats1/2* knockout mammary epithelium	TEADs	Luminal and basal cell-specific genes	YAP/TAZ cooperates with ERα and regulates the breast cell fate.	[49]
	ERα+ BC	TEADs, ER and MED1	E2/ERα-target genes	YAP/TAZ is required for estrogen-induced transcription and BC growth.	[50]
	ERα+ BC	TEADs	*ESR1*	YAP/TAZ is required for the transcriptional repression of *ESR1*.	[51]
	ERα+ BC	TEADs	*VGLL3*	YAP/TAZ-induced expression of *VGLL3* is required for NCOR2/SMRT complex-mediated transcriptional repression of *ESR1* gene.	[52]
	ERα+ BC	TEADs	*ESR1*	YAP inhibits ERα and ER+ BC growth by disrupting a TEAD-ERα signalling axis.	[53]
	Tamoxifen-resistant BC	TEADs	*CTGF*, *CYR61*, *Glut3* and *ERα*	YAP, CTGF and CYR61-induced transcriptional repression of *ERα* confers resistance to tamoxifen in BC.	[54]
	CDK4/6 inhibitors-resistant ER+ BC	TEADs	*CDK6*	Yap/TAZ-mediated CDK6 overexpression is required for *FAT1* or *RB1* loss-associated clinical resistance to CDK4/6 inhibitors in ER+ BC.	[58]
HER2+ BC	ILK-driven HER2+ BC	TEADs	YAP/TAZ target genes	YAP/TAZ activity is required for ERBB2 and ILK-driven mammary tumourigenesis.	[60–62]
	EphA2-driven HER2+ BC	TEADs	*SLC1A5* and *GLS*	YAP/TAZ-mediated transcriptions are required for EphA2-induced glutamine metabolism in HER2+ BC.	[63–65]
	Mechanical signalling-mediated oncogenic activation	/	/	YAP/TAZ activation accounts for the transcriptional responses downstream of oncogenic signalling.	[66]
	Lapatinib-resistant HER2+ BC	TEADs	*AREG*	YAP/TAZ-dependent transcriptions are required for rigid microenvironments-modulated lapatinib-resistance in HER2+ BC.	[70]
	Anti-HER2 treatment-resistant BC	TEADs	*Survivin* and *mTORC1*	YAP/TAZ-mediated transcriptions are required for mevalonate pathway-mediated resistance to HER2-targeted treatments.	[71]
	Trastuzumab-resistant BC	/	/	Over-expression of YAP/TAZ as well as HER-3 and HER2/HER3 heterodimer is synchronously remarkable in trastuzumab-resistant BC cells.	[72]
TNBC	*RASSF1A* inactivation-associated TNBC	TBX3, β-catenin and TEADs	*BIRC5*, *BCL2L* and *MYC*	β-catenin/TBX3-YAP/TEAD complex-mediated transcriptions are required for RASSF1A-loss or RASSF1C-activated BC invasive phenotypes.	[76]
	TNBC with chromosome 5q loss	TEADs	YAP/TAZ target genes	YAP/TAZ activity is required for *KIBRA* loss-derived metastatic and CSC-like behaviours.	[77]
	TNBC with *SYNPO2* downregulation	TEADs	*CYR61* and *CTGF*	Inhibition of YAP/TAZ activity is required for Synaptopodin-2 function in metastasis suppression of TNBC.	[78]
	AP1/YAP-expressed BC	AP1 and TEADs	AP1-YAP-TEADs target genes	YAP/TAZ/TEAD and AP-1 association at enhancers drives oncogenic growth of BC.	[79]
	ZEB1/YAP-expressed BC	TEADs and ZEB1	ZEB1-YAP-TEADs target genes	ZEB1 and YAP/TEAD interaction stimulates the BC cell aggressiveness.	[80]
	KLF5 overexpression-associated TNBC	TEADs and KLF5	KLF5-YAP-TEADs target genes	KLF5 and YAP/TEAD interaction stimulates the BC cell proliferation and migration.	[81, 82]
	Taxol-resistant TNBC cells	TEADs	*CYR61* and *CTGF*	YAP/TAZ and their downstream transcriptional targets *Cyr61* and *CTGF* are required for Taxol-resistance in BC cells.	[83–85]
	VEGF/NRP2 signalling-activated TNBC	TEADs	*Rad51*	YAP/TAZ-dependent *Rad51* expression contributes to the resistance of TNBC cells to cisplatin.	[86, 87]
	Recurrent and mesenchymal BC cells	TEADs	*CYR61*, *CTGF* and ferroptosis-associated genes	YAP/TAZ activity is required for DDR2-induced ferroptosis susceptibility of recurrent and mesenchymal BC cells.	[88]
BCSCs	Loss of Scribble or activation of EMT	TEADs	*CTGF* and *Survivin*	YAP/TAZ are required to sustain self-renewal and tumour-initiation capacities in BCSCs.	[24]
	Activation of serum response factor	TEADs and SRF	*IL6*, *THBS1*, *ETS1*, *DLL1* etc.	SRF-YAP association-induced *IL6* expression is critical for YAP-induced stemness in mammary epithelial cells and BC.	[93]
	/	TEADs	YAP/TAZ target genes	TAZ is required for metastatic activity and chemoresistance of BCSCs.	[95]
	ROR1-induced chemotherapy-resistance of BCSCs	TEADs	YAP/TAZ target genes	YAP activity is required for ROR1-mediated BCSC maintenance, self-renewal, and drug resistance.	[96–98]
TMEs	ECMs and CAFs	TEADs	*LM511*	TAZ regulates the transcription of *LM511* and the formation of a LM511 matrix, and then the LM511/α6Bβ1 association can contribute the self-renewal and tumour-initiation of BCSCs.	[103]
		TEADs	Cytoskeletal regulator genes, including *ANLN*, *DIAPH3* and *MYL9*	SRC-mediated YAP activation is required for CAFs to promote matrix stiffening, cancer cell invasion and angiogenesis in BC.	[104]
		TEADs	*IL11* and *IL15*	YAP-mediated breast stromal CAF activation can promote angiogenesis in a VEGF-independent manner.	[105, 106]
		TEADs	*CYR61*, *CTGF*, *BIRC5*, *ANLN*, *MYL9* etc.	YAP/TAZ activation in CAFs is responsible for CCM3 loss-induced BC metastasis.	[107]
		TEADs or β-catenin	ECM remodelling-associated genes	YAP/TAZ signalling is required for DKK3-mediated tumour-promoting activities of CAFs in BC.	[108]
		TEADs	*CTGF*, *GLS1* and *SLC1A3*	Mechanics-mediated YAP/TAZ activity is required for coordinating the metabolic crosstalk between CAFs and BC cells.	[109, 110]
	Adipocytes	TEADs	*Resistin*	TAZ-Resistin signalling promotes the BC growth and stemness.	[112]
	TIME	TEADs and p65	*HK2*	Activated YAP cooperates with TEAD-p65 to promote BC cell migration and metastasis.	[115]
		TEADs	YAP/TAZ target genes	YAP activity is required for *Cdh1* and *Pik3ca* mutations-induced immune-related ILC of the breast.	[118]
		TEADs	*PD-L1*	YAP/TAZ promote BC immune evasion through the transcriptional regulation of PD-L1.	[123]
		TEADs	*PD-L1* and *IL34*	TAZ-mediated transcriptions induce the proliferation and migration of TAMs and inhibited T cell infiltration, thereby forming an immunosuppressive microenvironment in TNBC.	[124]

A systematic review of YAP/TAZ-mediated oncogenic roles in various BC subtypes, BCSCs and TMEs.

Tamoxifen is a competitive inhibitor of estrogen by binding to ER, and it is widely used for ER+ BC treatment. Kim et al. found the expression of YAP and its downstream targets, including *CTGF* and *CYR61* were increased, while ERα was decreased in recurrent BC patients following tamoxifen treatment [54]. Further study showed that EGFR-induced YAP activation caused transcriptional repression of *ERα* by binding its promoter region, which eventually conferred resistance to tamoxifen in ER+ BC cells [54]. In addition, owing to the central role of estrogen-driven activation of CDK4/6 kinase in ER+ BC, multiple CDK4/6 kinase inhibitors, including palbociclib [55], ribociclib [56], and abemaciclib [57] have been developed for treating the advanced ER+ BC. However, CDK4/6 inhibitor resistance was also reported in BC patients. Li et al. have discovered that *FAT1* loss was associated with the clinical resistance to CDK4/6 inhibitors and YAP/TAZ-mediated upregulation of *CDK6* was a recurrent mechanism of resistance [58]. Taken together, all these discoveries indicate the clinical potential of YAP/TAZ as therapeutic targets for treating drug-resistance ER+ BC.

### HER2+ subtype

HER2+ BC is mainly characterised with *HER2/ERBB2* gene amplification or protein overexpression, which is occurred in 15-20% of total BCs. Therefore, inhibition of HER2-mediated signalling transduction is the major therapeutic strategy for this subtype of BC [59]. So far, multiple humanised monoclonal antibodies and tyrosine kinase inhibitors, such as trastuzumab, pertuzumab and lapatinib, have been widely applied for the clinical treatment of HER2+ BC patients [59]. Although these HER2-targeted drugs have dramatically improved the prognosis for BC patients, most of them ultimately experience disease progression, due to de novo or acquired resistance [59].

YAP/TAZ activation has been revealed to be associated with both tumourigenesis, metastasis, and drug resistance of HER2+ BC (Table 1 and Fig. 3). For example, integrin-linked kinase (ILK) is an integrin-associated protein that regulates several cell adhesion and integrin-mediated functions [60]. ILK LOF in ErbB2-expressing mammary epithelium dramatically reduces the cell proliferation, and mammary tumourigenesis [61]. Further investigation showed that genetic deletion of ILK in ErbB2-activated mammary tumours led to YAP inactivation, indicating an essential role for YAP activation in ErbB2-induced mammary tumourigenesis [62]. Similarly, EphA2, a receptor tyrosine kinase (RTK), is frequently overexpressed in human BCs [63]. Using a transgenic mouse model, Brantley-Sieders et al. reported that EphA2 promoted mammary adenocarcinoma tumourigenesis and metastasis by amplifying ErbB2 signalling [64]. Further investigations showed that EphA2 expression was positively correlated with YAP/TAZ nuclear localization in HER2+ BC, and YAP/TAZ-mediated glutaminolysis could predict enhanced metastatic potential and poor patient survival [65]. This study thus indicates that targeting YAP/TAZ holds great promise for EphA2-associated HER2+ BC treatment. More interestingly, Panciera et al. recently found that mechanical signalling from the microenvironment was essential for RTK-associated oncogenes, such as *EGFR* and *HER2*, to initiate mammary tumourigenesis, while YAP/TAZ activation accounted for the transcriptional responses downstream of these oncogenic signalling [66]. Furthermore, they also identified that Ras-related C3 botulinum toxin substrate 1 (RAC1) was a key mediator for coordinating RTK-associated cytoskeletal mechanosignaling [66]. All of these studies thus highlight the central roles of YAP/TAZ in HER2-mediated signalling transduction and tumourigenesis.

Multiple studies have revealed that anti-HER2 drug combinations, such as lapatinib plus trastuzumab regimen, can completely block the HER family receptor signalling, thereby improving the rates of pathologic complete response (PCR) in neoadjuvant clinical trials [67–69]. However, drug resistance still often developed. YAP/TAZ activation has been discovered to be involved in both lapatinib and trastuzumab resistance in HER2+ BC patients. Specifically, Lin et al. have reported that rigid microenvironments can modulate lapatinib resistance in HER2+ BC cells through activation of YAP/TAZ [70]. Downregulation of YAP/TAZ or inhibition of YAP/TEAD association increases the sensitivity to lapatinib treatment [70]. In addition, mevalonate pathway (MVA) is found to be activated in lapatinib+trastuzumab-resistant cells [71]. Further studies showed that YAP/TAZ-mTORC1-Survivin signalling was stimulated by MVA when HER2 activity was blocked [71]. These data thus suggest that inhibition of MVA is a potential therapeutic strategy to resensitize the tumours depending on MVA to progress on anti-HER2 therapies. More importantly, one clinical study also reported that high expression of YAP/TAZ could contribute to the occurrence of trastuzumab resistance, while YAP/TAZ inhibition reversed above resistance [72]. Taken together, all these preclinical and clinical findings highlight that targeting YAP/TAZ can contribute to the treatment of resistance to HER2-targeted therapies.

### TNBC subtype

TNBC is considered to be the most aggressive BC subtype, which always exhibits early recurrence and develops distant metastasis. TNBC accounts for about 15% of all BC patients; however, it causes about 30% of all BC-related deaths [73]. Due to the lack of HR expression and HER2 amplification, the above-mentioned endocrine and targeted therapies have no effect on TNBC treatment. Despite other targeted therapies, including PARP and programmed cell death-ligand 1 (PD-L1) targeting strategies, have been approved by FDA to treat BRCA-mutated or PD-L1-positive BC patients [74], the patients who can benefit from these therapies are still very limited. Anthracyclines and taxane-based chemotherapy agents are still the most commonly used chemotherapeutics for clinical treatment of TNBC. However, chemotherapy resistance remains a serious threat to patient survival [75].

Accumulating evidence has showed that YAP/TAZ activation in TNBC is associated with the tumour malignancy and drug-resistance in different contexts (Table 1 and Fig. 3). For example, many of the tumour suppressor genes, including *RASSF1A*, *KIBRA*, *SYNPO2*, are identified to be frequently down-regulated or lost in TNBC patients [76–78]. Meanwhile, YAP/TAZ activation is discovered to be required for the metastatic phenotypes caused by these gene losses. For instance, in a wide variety of sporadic malignancies, promoter methylation of the *RASSF1* gene is associated with tumour invasion and metastasis [76]. Vlahov et al. have showed that *RASSF1A* loss-induced phenotypes are driven by YAP-dependent transcription in BC cells [76]. In addition, using a TNBC mouse model with spontaneous loss of chromosome 5q, Knight et al. identified KIBRA as a metastasis suppressor [77]. Further investigations showed that KIBRA suppressed RHOA activation and the nuclear translocation of YAP/TAZ, which drove the metastatic and CSC-like behaviours of BC cells [77]. Moreover, multiple transcriptional factors, including AP1, ZEB1 and KLF5, have also been discovered to be co-recruited on the majority of YAP/TAZ-bound promoters or enhancers, thereby synergistically activating target genes to promote TNBC aggressiveness and metastasis [79–82], Therefore, disruption of YAP/TAZ-associated transcriptional complexes represents one of the most promising avenues for anti-YAP/TAZ therapeutic intervention.

In addition, YAP/TAZ activity is closely related to the chemotherapy resistance of TNBC. For example, YAP/TAZ-mediated transcriptions, including *CYR61* and *CTGF*, are responsible for taxol resistance in TNBC cells [83], while CDK1-induced YAP phosphorylation is related to the tumour cell apoptosis in the anti-tubulin drug response [84]. Moreover, one clinical study shows that the combined expression of YAP in tumour cells and non-lymphocytic stromal cells is associated with the reduced efficacy of anthracycline-taxane-based neoadjuvant chemotherapy in TNBC patients in terms of PCR rate [85]. The nuclear co-expression of YAP/TAZ thus may confer an increased risk of recurrence [85]. More interestingly, Rad51 is a central enzyme for homology strand exchange and repairing of damaged DNA [86]. Recent study has showed that YAP/TAZ-mediated *Rad51* transcription is required for VEGF-NRP2 signalling-mediated cisplatin resistance of TNBC cells, and overexpressing *Rad51* can rescue the defects in DNA repair upon inhibition of either VEGF-NRP2 or YAP/TAZ [87]. In addition, YAP/TAZ-mediated ferroptosis susceptibility is found to be activated in human recurrent mesenchymal BC cells with DDR2 protein overexpression [88]. Therefore, modulating YAP/TAZ-mediated ferroptosis in this context may have therapeutic potential for treating recurrent BC. Overall, all current studies highlight that YAP/TAZ represent a hub for the aggressiveness and recurrence of TNBC, and combinational inhibition of YAP/TAZ activity in TNBC treatment may serve as an effective strategy for avoiding tumour metastasis and overcoming drug resistance.

## YAP/TAZ-mediated oncogenesis in BCSCs

CSCs are defined as a part of the cell population, specifically endowed with self-renewal ability in vitro and tumour initiation potential in vivo [89, 90]. CD44^high^/CD24^low^ cells have been considered to be putative BCSCs, which can be isolated from both primary breast tumours and cancer cell lines [91]. Actually, early study in human embryonic stem cells has reported that YAP/TAZ are necessary for the self-renewal and pluripotency of stem cells [92]. In BC, Cordenonsi et al. initially showed that TAZ gain-of-function (GOF) endowed the self-renewal abilities to non-CSCs, and thus TAZ was considered to be a regulator of BC stemness [24]. Subsequently, Kim et al. found that YAP activation could induce a large number of MaSC signature genes, such as *IL6*, through cooperating with transcriptional factor SRF [93]. Furthermore, SRF-YAP-IL6 signalling was found to be enriched in basal-like BC patients and required for maintaining BC stemness [93]. Tumour metastasis and drug-resistance are the main causes of death in BC patients, and BCSCs have been suggested to be responsible for these BC cell behaviours [94]. Bartucci et al. have found that BCSCs exhibit a higher chemoresistance and migratory potential in vitro compared with the differentiated BC cells [95]. Specifically, TAZ LOF in BCSCs severely impairs metastatic colonisation and chemoresistance, while TAZ GOF in differentiated BC cells induces cell transformation and confers tumourigenicity and migratory activity [95]. This study thus demonstrates that TAZ is required for the metastatic ability and chemoresistance of BCSCs. ROR1, a tyrosine kinase-like orphan receptor, has been reported to play an important role in inducing stemness of BC cells [96, 97]. Zhang et al. have found that ROR1 expression is increased in BC cells treated with chemotherapy, and activation of YAP/TAZ is then proved to be responsible for ROR1-dependent chemotherapy resistance [98]. Together, all these findings highlight that targeting YAP/TAZ in BCSCs is of paramount importance in successfully preventing BC metastasis and relapse.

## YAP/TAZ-mediated oncogenesis in TMEs

Solid tumours are usually surrounded by a complex and heterogeneous microenvironment, which consists of acellular components, such as extracellular matrix (ECM), cytokines and other signalling molecules, and cellular components, including fibroblasts, endothelial cells, adipocytes, and diverse immune cells [99]. The TMEs have established complex interactions with the tumour cells during tumour development, and which can directly or indirectly contribute to maintaining the tumour cell survival. The TMEs are usually characterised by low oxygen, acid pH, increased interstitial pressure, fibrosis and immunosuppression [99].

YAP/TAZ activity is initially found to be regulated by cell-cell contact, which represents a proxy of the growth-suppressive tissue microenvironment [10, 26]. However, this effect is destroyed during tumourigenesis [99]. Subsequent research reveals the mechanical forces from neighbouring cells or from the surrounding ECM can affect the tension and organisation of the F-actin cytoskeleton, thereby playing a central role in the control of YAP/TAZ activity [100–102]. In BC, Chang et al. have found that TAZ regulates the formation of a LM511 matrix through transcriptionally regulating *LMa5* expression [103]. The activation of LM511/α6Bβ1 signalling can further contribute to the CSC-properties by activating TAZ [103]. These data thus highlight the positive feedback in BCSCs that involves in LM511/α6Bβ-mediated activation of TAZ and TAZ-mediated regulation of ECM. Cancer-associated fibroblasts (CAFs) can secrete high levels of cytokines and growth factors, and which are the major stromal residents in tumours [99]. YAP-induced gene expression in CAFs, including *ANLN*, *DIAPH3*, and *MYL9*, is required for breast CAFs to promote matrix stiffening [104]. Reciprocally, matrix stiffening can further enhance YAP activation in BC cells, thereby promoting cancer cell growth and invasion [104]. In addition, vascular endothelial growth factor (VEGF) activation has been proved to be a crucial signalling pathway in tumour angiogenesis [105]. Du et al. found that YAP-mediated breast CAF activation could promote the angiogenesis in the absence of VEGF signalling [106]. Moreover, IL11 and IL15 regulated by YAP in CAFs activated STAT3 signalling in endothelial cells, thereby stimulating angiogenesis resistance to anti-VEGF therapy [106]. Recent studies have also reported that mechanical-dependent YAP/TAZ signalling in CAFs is necessary for CCM3-associated metastatic spread of BC and DKK3-induced BC aggressive behaviours, respectively [107, 108]. Moreover, YAP/TAZ also participate in the metabolism regulation to coordinate the nutrient availability with tumour cell growth and survival [109]. Bertero et al. have linked ECM stiffening of both BC cells and CAFs to metabolic rewiring through a YAP/TAZ-dependent glutamate/aspartate crosstalk in the tumour niche [110]. Inhibition of metabolic reprogramming in either CAF or cancer cells can reduce tumour progression [110]. Taken together, all these findings highlight that YAP/TAZ-dependent ECM remodelling and mechanotransduction in both CAFs and BC cells function as central regulators of tumour cell proliferation, survival, metastasis and angiogenesis. Therefore, blocking of mechanical YAP/TAZ activation represents a therapeutic target for BC with CAF activation or increased ECM deposition. In addition, obesity has been proved to be a highly significant risk factor for BC development, and excessive adipose tissue in patients can stimulate cancer cell proliferation, metastasis, and avoidance of chemotherapy [111]. Gao et al. discovered that TAZ expression in adipocytes was stimulated by the free fatty acid/PPARγ axis upon dietary fat treatment [112]. Further investigations revealed that TAZ regulated the expression of numerous secreted proteins, such as Resistin, which in turn promoted BC growth and stemness [112]. This study thus supports that TAZ-Resistin signalling may serve as a potential therapeutic and diagnostic target for obesity-related BCs.

In the emerging era of tumour immunotherapy, understanding the characteristics of the tumour immune microenvironment (TIME) and the relationship between tumour cells and TIME is essential to develop strategies to improve immunotherapy response [113]. The immune-related cells in TMEs, including tumour-associated macrophages (TAMs), myeloid-derived suppressor cells (MDSCs), regulatory T cells (Tregs), and tumour infiltrating lymphocytes (TILs), have been reported to involve in the regulation of tumour immunity in BC. For example, macrophage-derived tumour necrosis factor α (TNFα) is a well-known cytokine that regulates the inflammatory processes in tumour development, while YAP activation has been demonstrated to be associated with the inflammatory microenvironment [114]. Specifically, Gao et al. found that TNFα-triggered YAP activation cooperated with TEAD-p65 to synergistically upregulate hexokinase 2 transcription, which in turn promoted BC cell migration and metastasis [115]. This study thus reveals an important role for macrophage-induced YAP activation in the process of inflammation-driven migration of BC. In addition, deletion of *Cdh1* and *Pik3ca* in mouse mammary epithelium can induce the formation of ILC with immune-related (IR) subtype [116], which is usually presented with immune cell infiltration and gene expression linked to lymphocyte and macrophage function [117]. An et al. have found that Yap-dependent transcription and signalling are activated in IR-ILC, and these tumour cells show sensitivity to Yap inhibitor [118]. More interestingly, PD-L1 is an immune checkpoint molecule that binds to its receptor PD-1 on T cells to suppress its antitumour activity [119]. Therefore, tumour PD-L1 status has been regarded as a biomarker for response to anti-PD-1/PD-L1 therapy [120–122]. In BC cells, one study has discovered that PD-L1 is a transcriptional target of YAP/TAZ, and pharmacologic inactivation of YAP/TAZ significantly inhibits *PD-L1* expression [123]. This study thus highlights the therapeutic potential of targeting YAP/TAZ to improve the efficiency for tumour immunotherapy. A similar study has also found that TAZ remodels the TIME in TNBC by directly regulating the transcriptions of *interleukin 34* and *PD-L1*, thereby inducing the proliferation and migration of TAMs and inhibiting T cell infiltration [124]. Moreover, combined targeting TAMs and immune checkpoint have shown therapeutic advantage for the treatment of TNBC [124]. Taken together, all these studies support that YAP/TAZ are multifunctional regulators in BC development, through coordinating both tumour cell behaviours and TIME remodelling.

## Targeting YAP/TAZ in BC

Given the above evidence that aberrant YAP/TAZ activation participates in a wide range of cellular events of BC development, directly targeting YAP/TAZ thus offers a potential therapeutic opportunity for BC treatment. Although attractive, YAP/TAZ inhibition may elicit toxicity, duo to their indispensability in tissue development and regeneration [20]. In addition, owing to the lack of a DNA-binding domain, YAP/TAZ require to cooperate with other DNA-binding proteins to modulate their transcriptional regulatory function in different context [125, 126]. TEAD family proteins seem to mediate most of the YAP/TAZ pro-tumourigenic functions [13]. Therefore, interfering with the formation of YAP/TAZ-TEAD complex or inhibiting TEADs directly can affect YAP/TAZ-associated transcriptional outcomes in tumours. Indeed, following this idea, numerous in vitro and in vivo preclinical studies have showed promise for tumour therapy. Besides, targeting either YAP/TAZ upstream regulators or their transcriptional outputs also shows a potent anti-tumour effect.

### Targeting YAP/TAZ-interacting transcriptional regulators

Here, we have mainly summarised the representative drugs targeting YAP/TAZ-TEAD complex based on different molecular mechanisms (Table 2). Verteporfin, an FDA-approved compound for treating macular degeneration, has been initially identified to block the interaction between YAP and TEAD, thereby inhibiting BC growth and metastasis in vitro and in vivo [127]. As with verteporfin, many other compounds, such as CA3 and CPD3.1 [128–130], are also gradually identified to be able to interfere with YAP/TAZ-TEAD-mediated activity, thereby inhibiting tumour cell growth. However, their target specificity and selectivity remain to be determined. VGLL4, a vestigial-like protein 4, is also found to be a tumour suppressor in human cancers via direct competition with YAP for binding TEADs [131, 132]. Therefore, a VGLL4-mimicking peptide called “super-TDU” has been designed and showed anti-tumour efficiency in vitro and in vivo [131, 132]. In addition, palmitoylation of TEAD transcription factors has been demonstrated to be required for their stability and the transcriptional output of the Hippo pathway [133, 134]. To this end, multiple drugs targeting TEAD palmitoylation have been developed and displayed anti-tumourigenic properties both in vitro and in vivo [135, 136]. Therefore, targeting TEADs-associated posttranslational modifications also provides a therapeutic potential for YAP/TAZ-TEAD-driven BC in the future. Up to now, there are three inhibitors that have entered the clinical stage I [137]. Among them, ION537 is an antisense nucleotide inhibitor, and for others targeting YAP-TEAD interaction, there is no chemical structure has been reported [137].

**Table 2:** Representative drugs targeting YAP/TAZ-interacting proteins.

Mechanisms	Drug name	Drug target	Pre-clinical evidence	Clinical phase	Main reference
YAP/TAZ-TEAD interaction	Verteporfin	YAP/TAZ-TEAD	Inhibited tumour cell proliferation, migration in vitro, and tumour growth in vivo.	/	[127]
	CPD3.1	YAP/TAZ-TEAD	Inhibited tumour cell proliferation and migration in vitro.	/	[128]
	CA3	YAP/TAZ-TEAD	Inhibited tumour cell proliferation with YAP high expression both in vitro and in vivo.	/	[129]
	Super-TDU	YAP/TAZ-TEAD	Inhibited YAP-mediated cell proliferation in vitro and tumourigenesis in animal models.	/	[131, 132]
TEAD palmitoylation	TED347	TEAD	Disrupted the YAP-TEAD interaction in cells and reduced the viability of patient-derived glioblastoma cell lines.	/	[135]
	MGH-CP1	TEAD	Inhibited TEAD-mediated transcription and intestinal epithelium expansion in vivo.	/	[136]
/	ION537	YAP/TAZ-TEAD	/	Phase I (NCT04659096)	[137]
	VT3989	YAP/TAZ-TEAD	/	Phase I (NCT04665206)	[137]
	IAG933	YAP/TAZ-TEAD	/	Phase I (NCT04857372)	[137]
YAP/TAZ-TEAD-interacting factors	SR-11302 or T5224	AP1	Inhibited YAP/TAZ-mediated gene transcription, oncogenic cell growth in vitro, and liver size regulation in vivo.	/	[139]
	Flavopiridol and NVP-2	CDK9	Inhibited YAP-dependent transcription and YAPS127A-driven liver growth in vivo.	/	[140]
	JQ1	BRD4	Inhibited YAP/TAZ-dependent transcription and cell proliferation in vitro.	/	[40]
	BAY-1238097	BRD4	Inhibited YAP/TAZ-dependent mammary tumour growth in vivo.	/	[40]

These drugs are mainly classified into three groups according to the molecular mechanisms behind the YAP/TAZ-TEAD inhibition.

In addition, many more of the YAP/TAZ-interacting partners are gradually being identified to regulate their functions in different contexts [125, 126]. Among these factors, AP-1 family members are the most representative factors that can cooperate with YAP/TAZ at the distal enhancers of YAP/TAZ-regulated genes through direct interaction, thereby synergistically driving oncogenic growth in multiple cancer types [138]. In this perspective, targeting AP1 directly or by inhibition of MEK/ERK signalling pathway may also offer the possibility to eliminate the YAP/TAZ-driven cancers. As stated, Koo et al. have showed that chemical AP-1 inhibitors (SR-11302 or T5224) can inhibit YAP/TAZ-mediated gene transcription, oncogenic cell growth in vitro, and liver size regulation in vivo [139]. Similarly, YAP/TAZ-mediated recruitment of general transcriptional cofactors at YAP/TAZ-regulated promoters/enhancers, including bromodomain-containing protein 4 (BRD4), CDK9, RNA polymerase II and the mediators, can also boost the expression of a host of growth-regulating genes [40, 140]. Therefore, targeting these factors also represents a potential therapeutic strategy for YAP-driven cancers. Indeed, Galli et al. have showed that inhibition of CDK9 can rescue YAPS127A-driven increase in liver size and target gene expression [140]. In addition, bromodomain and extraterminal domain (BET) inhibitors, such as JQ1 and BAY-1238097, have been proven to interfere with the activity of BRD4 [141]. Zanconato et al. showed that YAP/TAZ-addicted TNBC cells were sensitive to JQ1 treatment in vitro, while BAY-1238097 could efficiently reduce *Apc* loss-induced mammary tumourigenesis in vivo [40]. All these studies highlight that targeting YAP/TAZ-mediated transcriptional regulation may represent additional routes to interfere with YAP/TAZ function in BC.

### Targeting YAP/TAZ upstream regulators or downstream effectors

Extensive studies have highlighted the tumour suppressor role of the Hippo pathway in BC through regulating the localisation and stability of YAP/TAZ. Meanwhile, a large number of the upstream regulators of this pathway are gradually being discovered and described [125, 126]. Therefore, targeting these regulators to inhibit YAP/TAZ activity also represents a promising tumour-targeting strategy. For example, tyrosine kinase *SRC* is the first identified proto-oncogene in mammalian cells, and it has been reported to be frequently overexpressed or aberrantly activated in BC patients [142]. Specifically, SRC-mediated YAP phosphorylation is required for BC-associated fibroblasts to promote matrix stiffening, BC cell invasion and angiogenesis [104]. In BC, SRC-dependent YAP activation can promote the expansion of chemoresistant CSCs [143], as well as YAP/β-catenin-mediated transcription and invasion [76]. Moreover, SRC also can directly phosphorylate LATS1 on multiple tyrosine residues, thereby attenuating LATS kinase activity and enhancing YAP-associated BC growth [144]. All these findings highlight that targeting SRC kinase may have multiple inhibitory effects on YAP1 activity to suppress BC. Indeed, numerous preclinical and clinical data have demonstrated that Dasatinib, an orally available small molecule targeting multiple Src family kinases [145], in combination with other drugs could interfere with the tumour progression and reverse drug resistance in selected BC subtypes [146, 147]. Typically, a phase II trial combining Dasatinib with trastuzumab and paclitaxel has shown to be active with an objective response rate of almost 80% in HER2+ metastatic BC patients [146]. In TNBC, although a phase II study showed that single-agent Dasatinib had limited activity in unselected patients [147], the recent preclinical data proved that the combination of Dasatinib with paclitaxel synergistically reduced cell viability of pac-resistant cells in vitro and significantly inhibited breast tumour growth in vivo [148, 149]. Therefore, combining Dasatinib with chemotherapy may be worth pursuing in the clinical setting.

Considering that YAP/TAZ function as the important transcriptional co-regulators in BC development and progression, targeting YAP/TAZ-dependent downstream effectors in a defined context, including metabolic enzymes, kinases, ligands, etc, also represents a very promising therapeutic strategy. For example, YAP-mediated *CDK6* overexpression has been reported to be a key driver for the resistance to CDK4/6 inhibitors in BC patients, implying that more potent inhibitors of CDK6 may be valuable as strategies for overcoming drug resistance in clinical trials [58]. In addition, *PD-L1* has recently been identified as a bona fide transcriptional target of YAP/TAZ in human BC cells, suggesting that YAP/TAZ-dependent transcriptional regulation may direct the BC immune evasion [123]. Therefore, targeting YAP/TAZ as a monotherapy or in combination with PD-L1-targeted checkpoint inhibitors may provide clinical benefit during BC treatment.

## Conclusion and future perspectives

Given all the discoveries we have summarised in this review, there is no doubt that YAP/TAZ play a central role in BC development and malignancy. Therefore, targeting YAP/TAZ represents a very promising strategy for BC treatment. Indeed, extensive studies have demonstrated that directly targeting YAP/TAZ or their binding partners can inhibit the tumour growth both in vitro and in vivo. However, all these compounds remain in preclinical testing or the early stages of clinical trials. More importantly, considering the important roles of YAP/TAZ in the maintenance of homoeostasis tissue regeneration, using systemic inhibition of YAP/TAZ warrants caution as it may produce severe side effects and toxicity. To this end, targeting YAP/TAZ-mediated signalling transductions in a BC-specific context, including their upstream regulators, downstream effectors, and their interacting partners in transcriptions, may represent a more reasonable strategy for BC treatment. Therefore, further elucidating the mechanisms of YAP/TAZ activation and YAP/TAZ-mediated transcriptions in specified human BC subtypes and contexts, will enable us to develop more precise therapeutic targets and strategies in the future.

## Data Availability

The datasets used and analysed in this study are available from the corresponding author on reasonable request.
